# Histidine-rich calcium binding protein promotes gastric cancer cell proliferation, migration, invasion and epithelial-mesenchymal transition through Raf/MEK/ERK signaling

**DOI:** 10.7150/jca.68403

**Published:** 2022-01-04

**Authors:** Chao Wang, Chuanfu Ren, Qiongyuan Hu, Xiaofei Shen, Meng Wang, Zhi Yang, En Xu, Xingzhou Wang, Zijian Li, Heng Yu, Qingzhao Feng, Liang Zhang, Xuefeng Xia, Song Liu, Wenxian Guan

**Affiliations:** 1 Department of General Surgery, Nanjing Drum Tower Hospital, The Affiliated Hospital of Nanjing University Medical School, Nanjing 210008, China; 2 Department of General Surgery, Nanjing Drum Tower Hospital Clinical College of Nanjing Medical University, Nanjing 210008, China.

**Keywords:** HRC, Calcium homeostasis, EMT, Gastric cancer, Metastasis

## Abstract

Histidine-rich calcium binding protein (HRC) is a new type of Ca^2+^ homeostasis regulator, which acts as a nonnegligible role in regulating intracellular calcium homeostasis. Here, we demonstrated that HRC expression was upregulated in human gastric cancer (GC) samples, and its expression level was closely correlated with the overall survival (OS) rate of GC patients and the malignant potential of GC cell lines. Knockdown of *HRC* inhibited migration, invasion, and proliferation of GC cell lines *in vitro*, while *HRC* overexpression promoted GC cell migration, invasion, and proliferation* in vitro*, as well as the growth of subcutaneous tumors and peritoneal tumors *in vivo*. In terms of the mechanism, knockdown of *HRC* reduced the intracellular calcium ion level and the CaM protein level. Through cell function experiments, we found that HRC regulated the Raf/MEK/ERK pathway through Ca^2+^/CaM signaling and ultimately affected the epithelial‑mesenchyme transition (EMT) of GC. In summary, we revealed that HRC represents a potential target for GC treatment.

## Introduction

Gastric cancer (GC) is a typical malignant tumor that originates from the gastric mucosal epithelium 1. Currently, there are approximately one million new cases of GC in the world every year, and the mortality of GC in China accounts for approximately 50% of the world's total 2, 3. Early diagnosis of GC is one of the key factors for good patient prognosis. How to diagnose early GC is a hot research field of GC that many scholars pay attention to. Therefore, in-depth exploration of the molecular mechanism changes in the pathogenesis of GC and the discovery of potential biomarkers has far-reaching significance for the early diagnosis and precise treatment of patients with GC.

Histidine-rich calcium binding protein (HRC) is a new type of sarcoplasmic reticulum (SR) Ca^2+^ uptake, storage and release regulator, which has critical roles in the regulation of intracellular Ca^2+^ homeostasis 4. It is universally acknowledged that Ca^2+^ plays a nonnegligible role in tumorigenesis and development, including in GC 5-7. Although studies have shown that HRC overexpression reduces the rate of Ca^2+^ uptake in the SR, it also increases Ca^2+^ extrusion by regulating the level of Na^+^/Ca^2+^ exchange protein (NCX) 8, 9. This suggests that HRC's regulation of the intracellular Ca^2+^ concentration is relatively complicated. HRC increases [Ca^2+^]_i_ and promotes tumor metastasis in hepatocellular carcinoma (HCC) through Ca^2+^/calmodulin (CaM) signaling 10. Moreover, studies have reported that HRC promotion of the growth of HCC can be mediated by the MEK/ERK pathway and endoplasmic reticulum stress 11, 12. Recently, Lu et al. found that vitamin D inhibits lung cancer tumor growth, migration, and proliferation by downregulating HRC 13. However, the function and mechanism(s) behind the role of HRC in GC are still unclear.

Epithelial-mesenchymal transition (EMT) is a developmental process that promotes the transformation of epithelial cells into mesenchymal cells with migration ability 14. The abnormal activation of EMT plays an important role in the occurrence and development of tumors, especially GC, and correlates markedly with many characteristics of cancer, particularly metastasis 15-17. During EMT, epithelial cells break away from the connection with neighboring cells, and apical and basal polarity changes, displaying the characteristics of mesenchymal cells. Changes in cellular protein levels are mainly related to the increased expression of Snail, Slug, Vimentin, N-cadherin, and other proteins, while the expression of the epithelial cell marker E-cadherin decreases 18. EMT is regulated in many ways; therefore, cells that have undergone EMT in different tumor types or even at different stages of the same tumor, although morphologically similar, play different roles in metastasis 19. Our research group has been committed to the study of the mechanism of GC EMT regulation, and it is of great significance to explore the role of different oncogenes in the GC EMT process.

In the present study, our results revealed the effects of HRC on the invasion, proliferation, and migration of GC cells and further determined the potential molecular mechanisms* in vitro* and *in vivo*. In addition, our results also revealed that the knockdown of *HRC* inhibited the EMT in GC and further led to a reduction in cell proliferation, invasion, and migration. Studies on the mechanism of this effect indicated that HRC regulated the level of Raf/MEK/ERK phosphorylation by changing the intracellular Ca^2+^ concentration and CaM protein level. Overall, our findings demonstrated that HRC could serve as a potential therapeutic target in GC.

## Materials and Methods

### Clinical GC tissues

GC tissue samples were obtained from Nanjing Drum Tower Hospital (Nanjing, China). A total of seven patients with gastric cancer (GC) from January 2021 to June 2021 at the Department of Gastrointestinal Surgery, Nanjing Drum Tower Hospital, The Affiliated Hospital of Nanjing University Medical School, were enrolled in our study. These patients all had all locally advanced gastric cancer and underwent radical gastrectomy and D2 lymph node dissection. None of the patients received chemotherapy or radiotherapy before surgery, and GC was histopathologically confirmed. The GC tissues and the corresponding normal mucosa tissues at least 5 cm from the outer tumor margin were collected from all patients immediately after resection. This study was approved by the Ethics Committee of Nanjing Drum Tower Hospital. All patients provided written informed consent.

### Tissue microarray and immunohistochemistry (IHC) analysis

A human GC tissue microarray (HStmA180Su09, IRB approval number: SHYJS-CP-1607004) was purchased from Shanghai Outdo Biotech Co. Ltd. (Shanghai, China). Anti-HRC antibody (Sigma-Aldrich, St. Louis, MO, USA; SAB1303636) was used to incubate with the sections. Then we divided the specimens into four groups based on the intensity of staining: 3 (strong staining), 2 (moderate staining), 1 (weak staining), and 0 (negative staining), while the percentage of tumor cells was graded as follows: 4 (> 75%), 3 (51-75%), 2 (26-50%), and 1(≤ 25%). The staining intensity score multiplied by the proportion grade was the total score of the specimen. We defined a sample score ≥ 7 as high expression; otherwise, it was defined as low expression.

### Cell culture

MGC-803, MKN-45, AGS, and GES-1 cells were obtained from Shanghai Zishi Biotech Co., Ltd. (Shanghai, China). These cells were maintained in DMEM (Gibco, Grand Island, NY, USA) containing 10% fetal bovine serum (FBS; Gibco).

### Western blotting

The detailed experimental procedures were as described previously 20. Briefly, RIPA lysis buffer was used to extract proteins from cell samples and tissues. Then we separated equal amounts of lysate protein by SDS-PAGE, and then transferred them onto a PVDF membrane. The following primary antibodies were used: HRC (Sigma-Aldrich, SAB1303636), c-Raf (Affinity Biosciences, Cincinnati, OH, USA; AF6065), phosphorylated (p)-c-Raf (Affinity Biosciences, AF3065), MEK (Affinity Biosciences, AF6385), p-MEK (Affinity Biosciences, AF8035), ERK (Affinity Biosciences, AF0155), p-ERK (Affinity Biosciences, AF1015), N-cadherin (Cell Signaling Technology, Danvers, MA, USA; #13116S), Calmodulin (Cell Signaling Technology, #S35944), Vimentin (Cell Signaling Technology, #5741S), E-cadherin (Cell Signaling Technology, #3195S), Snail (Cell Signaling Technology, #S3879), Slug (Cell Signaling Technology, #9585S), and GAPDH (Abcam, Cambridge, MA, USA; ab9485).

### Small interfering RNA (siRNA) transfection

AGS and MKN-45 cells were transfected with HRC-specific siRNAs (siRNA#1, 5'-GGUCAAGGAUAGAAGCCAUTT-3', 5'-AUGGCUUCUAUCCUUGACCTT-3'; siRNA#2, 5'-CCCUAGAGACCAUCCAGAUTT-3', 5'-AUCUGGAUGGUCUCUAGGGTT-3') and control siRNAs. All siRNAs were purchased from Shanghai GenePharma Company (Shanghai, China). We seeded the cells (2×10^5^ per well) in a six-well plate and then transfected the cells with siRNA (1-2 µg) encapsulated by the interferin siRNA transfection reagent (Polyplus, Berkeley, CA, USA). HRC knockdown efficiency was assessed by Western blot analysis.

### Lentivirus transductions

Short hairpin RNA targeting *HRC* (shHRC) lentiviral particles and lentiviral particles containing human full-length *HRC* cDNA (oe-HRC) were obtained from OBiO Technology Corp., Ltd. (Shanghai, China). Briefly, we first infected the cells with lentiviral particles and then used puromycin (1 mg/mL) (Thermo Fisher Scientific, Waltham, MA, USA) to screen for 3 weeks based on the manufacturer's protocol.

### Gene set enrichment analysis (GSEA)

Gene sets of gene ontology for biological processes and KEGG pathways were downloaded from the Molecular Signatures Database (MSigDB, http://www.gsea-msigdb.org/) or KEGG PATHWAY Database (https://www.kegg.jp/). RNA sequencing data of 375 GC tissues in the TCGA-STAD cohort were downloaded from UCSC Xena (https://xenabrowser.net/datapages/). To identify the gene sets that correlated with *HRC*, we first calculated the correlation coefficient between the expression of *HRC* and all the other genes. All genes were then preranked according to the correlation coefficient and subjected to GSEA utilizing the R package “fgsea 1.18.0”, which was also used to plot the results.

### Wound healing assay

When the density of AGS and MKN-45 cells reached 100% in a 6-well plate, we used a sterile pipette tip to scratch the cell surface. We took pictures of the wound at 0 and 48 hours through a microscope. The cell migration rate was calculated using the following formula = (the initial gap (0 h) - gap (48 h))/the initial gap (0 h) × 100%.

### Transwell assay

For cell migration assays, we added 500 μl DMEM containing 10% FBS to each well of a 24-well plate, resuspended 5 × 10^4^ cells in 200 μl DMEM without FBS, and finally seeded these cells into the upper chamber of the Transwell apparatus (Costar, Corning Inc., Corning, NY, USA). For the invasion assay, 500 μl of DMEM containing 10% FBS was added to the 24-well plates per well. Then we applied Matrigel matrix to the upper chamber of the Transwell apparatus, resuspend 1 × 10^5^ cells in 200 μl DMEM without FBS and seeded them into the upper chamber. After 24 hours, we fixed these Transwell inserts with methanol at room temperature for 15 minutes, stained them with 0.1% crystal violet at room temperature for 15 minutes, washed them with PBS three times and observed them under a microscope.

### Colony formation assay

A total of 600 GC cells treated with* HRC* siRNA or lentivirus overexpressing* HRC* were seeded in a 6-well plate and incubated for 14 days. Then we fixed these cells with methanol at room temperature for 15 minutes and then stained them with 0.1% crystal violet at room temperature for 15 minutes. Finally, we photographed and recorded cell colonies containing more than 50 cells.

### Intracellular Ca^2+^ measurement and Ca^2+^ concentration determination

We used Fluo-4/AM fluorescent indicator (Beyotime Institute of Biotechnology, Jiangsu, China) to detect intracellular calcium levels according to the instruction manual. Briefly, the culture medium was removed and the cells were washed three times with PBS. Then we added 1.5 ml Fluo-4/AM working solution to each well of the six-well plate and incubated at 37 °C for 45 minutes for fluorescent probe loading. Then, the cells in the cells were washed with PBS three times and incubated for 30 minutes after washing. Finally, we used a fluorescence microscope to detect the fluorescence of Fluo-4 to determine the changes in the intracellular calcium concentration.

For intracellular Ca^2+^ concentration determination, we used a calcium colorimetric assay kit (#MAK022, Sigma-Aldrich) based on the manufacturer's protocol. In brief, each group of GC cells cultured in a 6-well plate was washed with PBS to remove residual medium. Then, we placed the cells on ice and added 150 μl of the cells to the wells of a 6-well plate, after which we homogenized the cells by ultrasonication. We centrifuged the homogenate at 12000 x *g* for 10 minutes and then removed 50 μl of the supernatant. We added 90 μl of chromogenic reagent to each well containing the standard and mixed gently, and then added 60 μl of calcium assay buffer to each well and mixed gently. Then, we incubated the cells at 37 °C in the dark for 8 minutes. Finally, we measured the absorbance of the sample to be tested at 575 nm (A575). The following formula was used to calculate the intracellular Ca^2+^ concentration: C = Sa/Sv (C, concentration of calcium in the sample; Sa, amount of calcium in the unknown sample (μg) from the standard curve; Sv, sample volume (μl)).

### Animal experiments

For the subcutaneous tumor growth assay, 5 × 10^6^ AGS-shHRC (GC cells silenced for *HRC* using a shRNA) and AGS-OE HRC (GC cells overexpressing *HRC*) in 300 µL of PBS were injected subcutaneously into the left iliac skin of the male nude mice. The mice were euthanized on day 25, and the xenografts were analyzed.

For the peritoneal dissemination assay, 3 × 10^6^ AGS-shHRC in 400 µL of PBS were injected into the male nude mouse peritoneal cavity. Peritoneal metastasis (PM) nude mice were analyzed when the mice were euthanized at 15 days post-injection. All animal procedures were approved by the Ethics Committee of Nanjing Drum Tower Hospital.

### Statistical analysis

GraphPad Prism software version 7.04 was used for all statistical analyses**.** Survival curves were generated by the Kaplan-Meier method and were compared using a log-rank test. The differences among the experimental groups were analyzed using Student's t test. Pearson's χ^2^ test was used to compare the frequencies of categorical variables. P < 0.05 was considered to be statistically significant.

## Results

### HRC is overexpressed in GC

**Fig 1:**
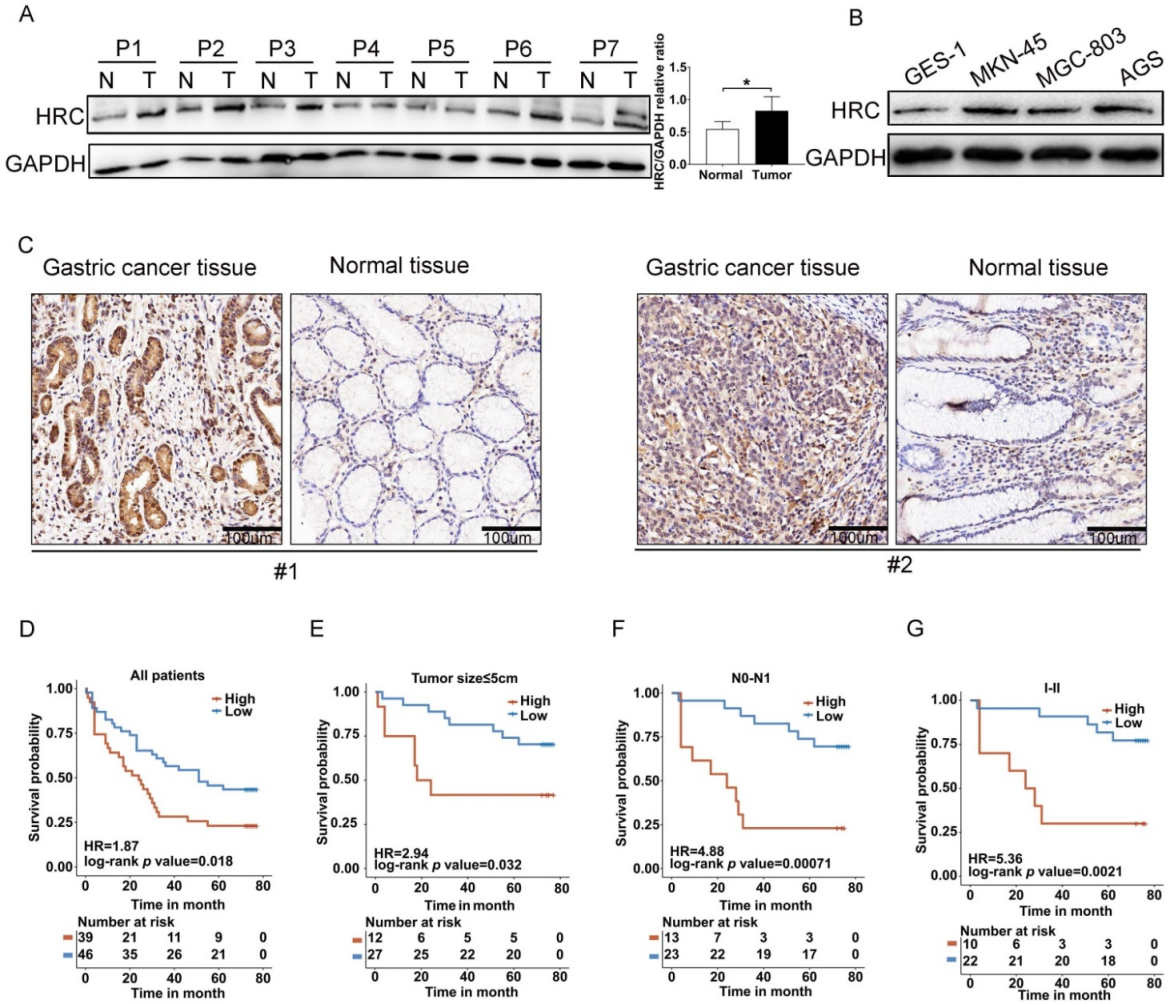
** HRC is overexpressed in GC cell lines and tumor tissues.** (A) Adjacent normal (N) and tumor (T) tissues of patients with gastric cancer (GC) were collected and lysates were subjected to western blotting for HRC expression. (B) Western blot analysis of HRC expression in the indicated cell lines. (C) Representative immunohistochemical staining of HRC in adjacent nontumor tissues and GC tissues. (D-G) Kaplan-Meier analysis of overall survival (OS) of patients with GC according to HRC expression in all patients (D), patients with tumor size ≤ 5 cm (E), patients with N0-N1 stage tumors (F), and patients with clinical stage I+ II tumors (G). HRC, histidine-rich calcium binding protein. Data are shown as the mean ± SD; *P < 0.05, based on Student's t-test.

To verify the expression of HRC protein in GC, we first assessed its expression in the tumorous and adjacent normal gastric mucosal tissues from seven GC patients (Fig. 1A), in the GES-1 cell line (normal gastric epithelial cell line), and in GC cell lines (AGS, MKN-45, and MGC-803) (Fig. 1B) using western blotting. We found that compared with the corresponding normal gastric epithelial cells or normal tissues, HRC showed higher expression in GC cell lines or GC tissues. IHC staining results also verified the high expression of the HRC protein in GC tissues (Fig. 1C). To investigate the relationship between HRC and the prognosis of patients with GC, we used the anti-HRC antibody for GC tissue microarray-based IHC. We determined the relationship between HRC expression and clinicopathological variables and summarized them in Table 1. Our results showed that the expression of HRC was correlated with tumor size and T stage. Moreover, GC patients with high HRC expression had significantly lower overall survival (OS). We also analyzed OS based on other key clinicopathological factors according to HRC levels. There were significant differences in tumor size, N0-N1, and clinical stage I+ II tumors (Fig. D-G). These results suggested that HRC is frequently upregulated in GC samples and is closely related to the survival of GC patients.

### HRC regulates malignant phenotypes of GC cells in vitro

**Fig 2:**
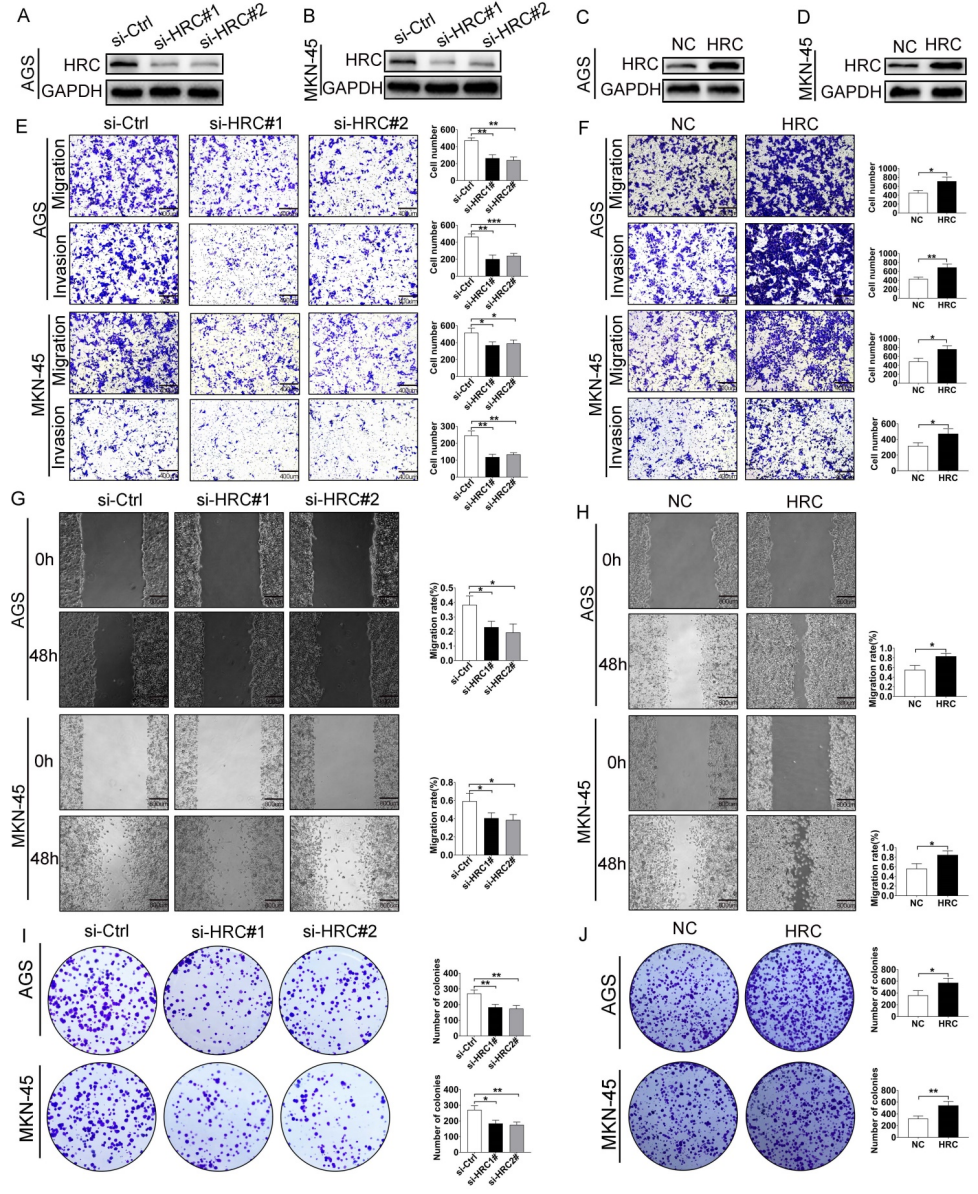
** HRC promotes GC cell migration, invasion and proliferation in vitro.** (A-B) Western blotting analysis of HRC expression in *HRC* siRNA-treated GC cells (AGS cells and MKN-45 cells). (C-D) GC cells (AGS cells and MKN-45 cells) transfected with *HRC-*overexpressing lentivirus. The overexpression of HRC in GC cells was detected by western blotting. (E) Knockdown of *HRC* reduced the migration and invasion of AGS and MKN-45 cells, as assessed using Transwell assays with/without Matrigel. (F) Overexpression of *HRC* increased the migration and invasion of AGS and MKN-45 cells, as assessed using Transwell assay with/without Matrigel. (G) Knockdown of* HRC* decreased AGS and MKN-45 cell migration, as measured using wound healing assays. (H) Overexpression of *HRC* increased AGS and MKN-45 cell migration, as measured by wound healing assays. (I-J) The number of cell colonies in AGS and MKN-45 cell lines was determined using a colony formation assay. HRC, histidine-rich calcium binding protein; GC, gastric cancer; siRNA, small interfering RNA. Data are shown as the mean ± SD; *P < 0.05, **P < 0.01, ***P < 0.001, based on Student's t test.

To further explore the biological functions of HRC, we transfected control siRNA (si-Ctrl) and HRC-siRNA (si-HRC#1 and si‑HRC#2) into AGS (Fig. 2A) and MKN-45 cells (Fig. 2B). After 72 hours of treatment, the levels of the HRC protein, cell migration, invasion, and clone formation were determined. We demonstrated that the knockdown of *HRC* effectively downregulated the level of the HRC protein in the two cell lines (Fig. 2A and 2B). In addition, the knockdown of *HRC* significantly inhibited the migration (Fig. 2E), invasion (Fig. 2E), wound healing (Fig. 2G), and colony formation (Fig. 2I) of the GC cells compared with the control group. When AGS and MKN-45 GC cell lines were treated with HRC-overexpressing lentivirus (Fig. 2C-D), GC cell migration (Fig. 2F), invasion (Fig. 2F), wound healing (Fig. 2H), and colony formation (Fig. 2J) were enhanced.

### GSEA enrichment analysis of HRC

**Fig 3:**
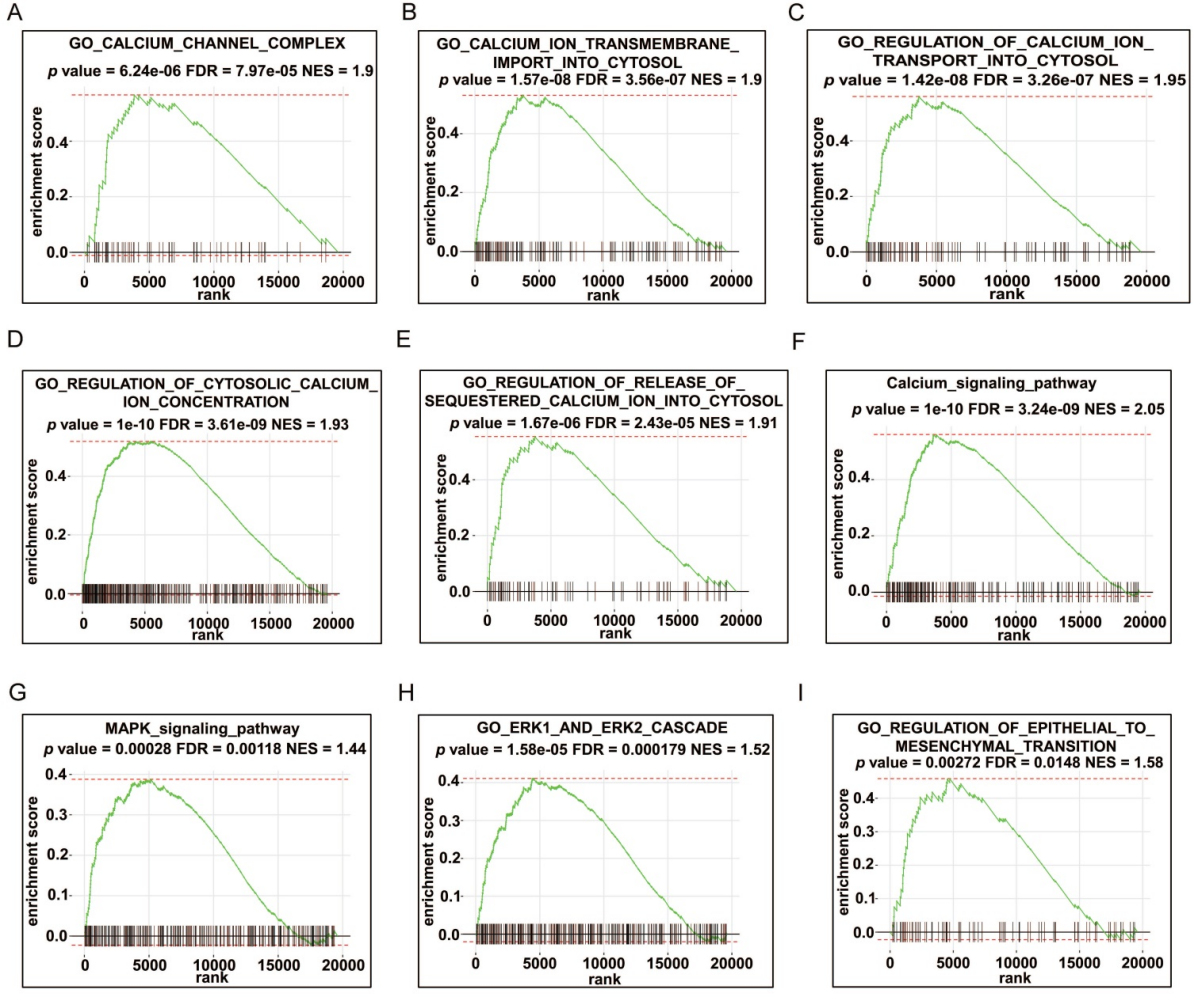
** GSEA enrichment analysis of HRC.** (A-I) GSEA was performed using the TCGA-STAD cohort according to the correlation coefficient between the expression of HRC and all the other genes. HRC, histidine-rich calcium binding protein; GSEA, gene set enrichment analysis

To predict the possible biological processes that involve HRC, we performed GSEA in the TCGA‑STAD cohort according to the correlation coefficient between the expression of *HRC* and all the other genes. The results showed that abundant biological processes were closely connected to calcium regulation, such as “calcium channel complex”, “calcium ion transmembrane import into cytosol”, and “regulation of cytosolic calcium ion concentration” et al. (Fig. 3A-F, Fig S1A-E). To investigate the specific mechanism of the phenotypic change caused by *HRC* knockdown, we employed the GSEA algorithm again to identify the pathways that correlated with *HRC* expression. We found that the MAPK/ERK signaling pathway was mainly enriched (Fig. 3G-H, Fig. S1F). The GSEA results of *HRC* also indicated that HRC is involved in the EMT process (Fig. 3I, Fig. S1G). Therefore, we hypothesized that HRC plays a pivotal role in the biological process of GC cells.

### HRC induces GC cell EMT via Raf/MEK/ERK signaling

**Fig 4:**
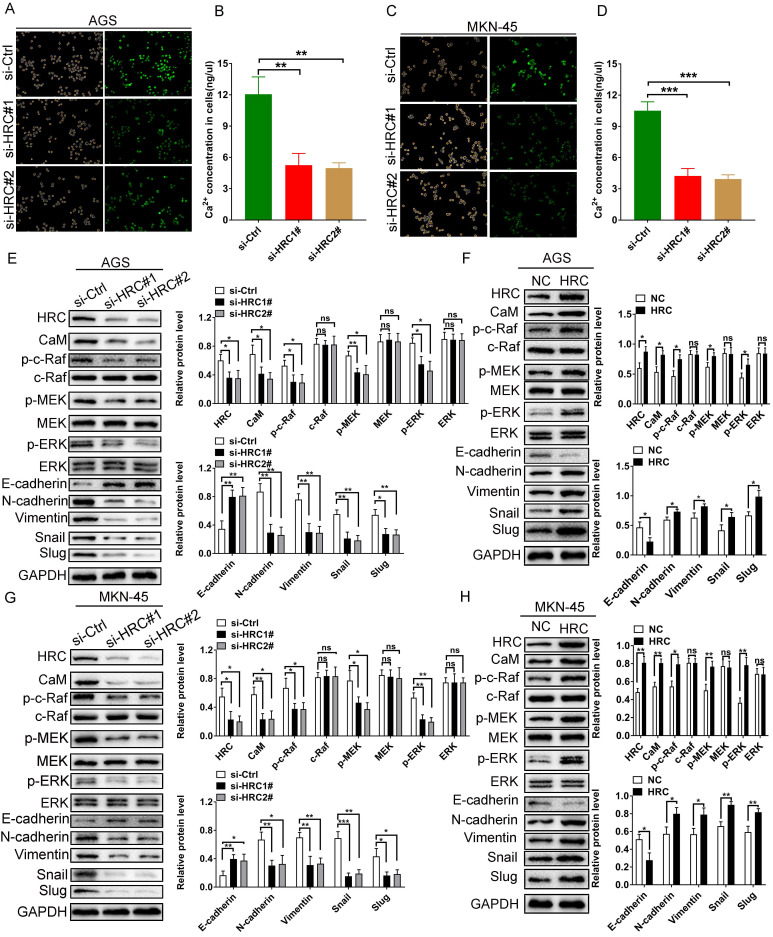
** HRC induces GC cell EMT via Ca^2+^/CaM signaling.** (A) AGS cells were transfected with control-siRNA or HRC-siRNA and stained with Fluo‑4/AM. (B) Knockdown of *HRC* reduced the intracellular Ca^2+^ concentration in AGS cells. (C) MKN-45 cells were transfected with control-siRNA or HRC-siRNA and stained with Fluo‑4/AM. (D) Knockdown of *HRC* reduced the intracellular Ca^2+^ concentration in MKN-45 cells. (E-H) Levels of p-c-Raf, c-Raf, p-MEK, MEK, p-ERK, ERK, E-cadherin, N-cadherin, Vimentin, Snail and Slug proteins in AGS and MKN-45 cells were analyzed by western blot. HRC, histidine-rich calcium binding protein; siRNA, small interfering RNA; c-Raf, Raf-1 proto-oncogene, serine/threonine kinase; p-c-Raf, phosphorylated c-Raf; MEK, MAPK/ERK kinase 1; p-MEK, phosphorylated MEK; ERK, extracellular regulated kinase; p-ERK, phosphorylated ERK; CaM, calmodulin. Data are shown as the mean ± SD; ns: no significant difference; *P < 0.05, **P < 0.01, ***P < 0.001, based on Student's t test.

Bioinformatic analysis results showed that HRC is involved in regulating the calcium ion concentration in GC cells. Therefore, we explored whether HRC can regulate the calcium ion concentration in GC cells. Then we used the Fluo‑4/AM Ca^2+^ fluorescence probe to identify changes in the distribution of Ca^2+^ in GC cells *in vitro*. The results showed that the silencing of *HRC* by its specific siRNA reduced the intracellular concentrations of Ca^2+^ in GC cells (Fig. 4A and 4C). To further quantify the changes in calcium ion concentration, a calcium colorimetric analysis kit was used to determine the difference between the concentration of Ca^2+^ in *HRC* knockdown cells and that in the control cells. The results demonstrated that the intracellular Ca^2+^ concentration in the *HRC* knockdown group was lower than that in the control group (Fig. 4B and 4D).

Furthermore, our experimental results also verified that knockdown of *HRC* reduced the abundance of the CaM protein in GC cells, while *HRC* overexpression increased the level of CaM (Fig. 4E-H). The Raf/MEK/ERK pathway is involved in the development of aggressive GC phenotypes and the EMT process 21, 22, and Ca^2+^/CaM is also essential for the activation of the Raf/MEK/ERK pathway 23, 24. Therefore, to investigate whether HRC affects the activation of Raf/MEK/ERK signaling in GC cells, we evaluated the levels of phosphorylated Raf, MEK, and ERK.

Consistent with the results of bioinformatics analysis, the levels of phosphorylated Raf, MEK and ERK in the *HRC* knockdown groups were significantly reduced compared with the control group, while they increased in the *HRC* overexpression groups (Fig. 4E-H). Subsequently, we detected the relevant indicators of EMT using western blotting, and the results revealed that compared with those in the control group, the levels of N-cadherin, Vimentin, Snail, and Slug in the* HRC* knockdown group were reduced significantly, while the level of E-cadherin increased. In addition, overexpression of *HRC* promoted the EMT in GC cells. These data indicated that HRC regulates GC cell EMT through the Raf/MEK/ERK pathway.

### The role of HRC in GC affects Raf/MEK/ERK signal activation by regulating the Ca^2+^/CaM pathway

**Fig 5:**
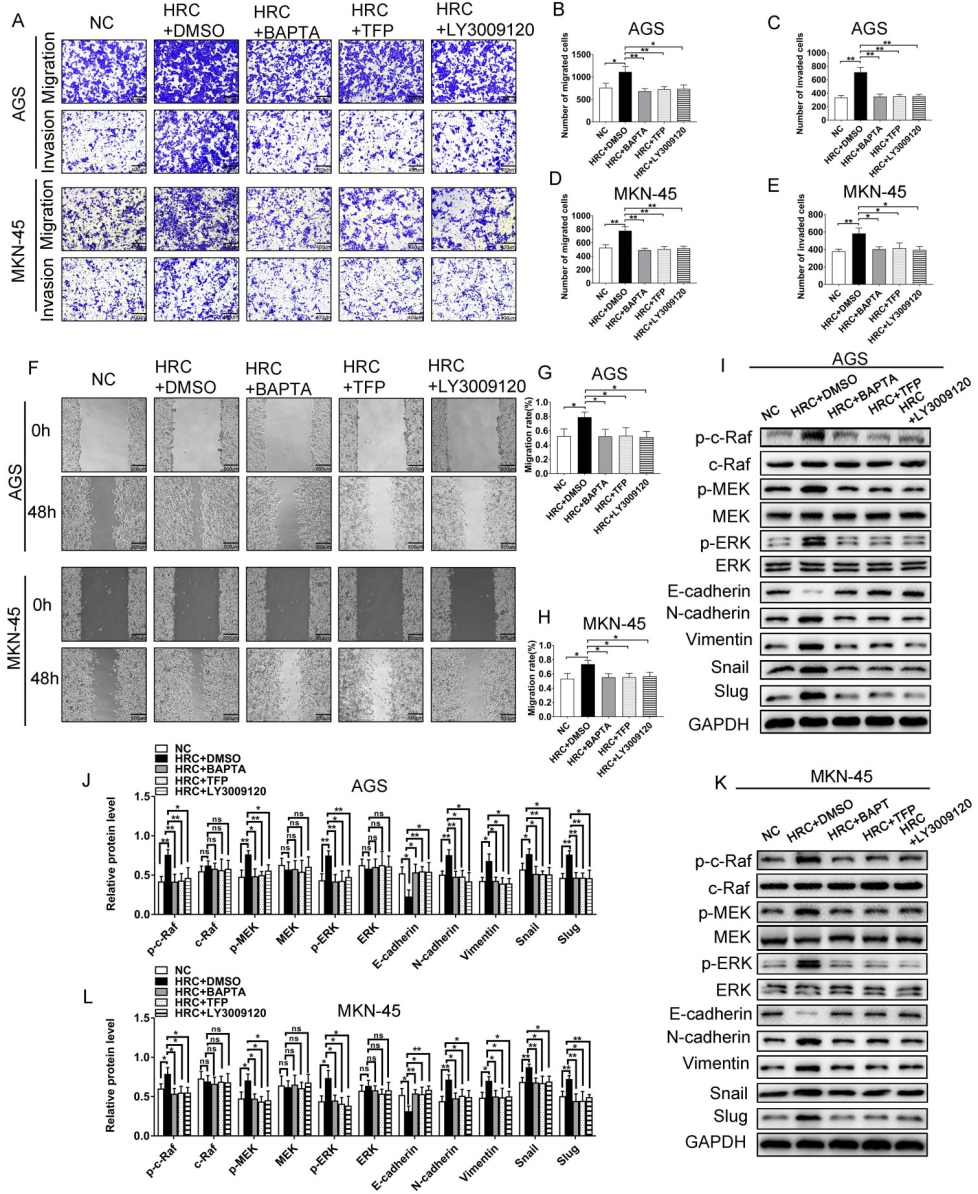
** The role of HRC in GC affects Raf/MEK/ERK signal activation by regulating the Ca2+/CaM pathway.** (A-E) Transwell assay with/without Matrigel showing that pretreatment with the Ca^2+^ inhibitor BAPTA/AM, the CaM inhibitor TFP, and the pan-Raf inhibitor LY3009120 abolished HRC-enhanced cell migration and invasion in AGS and MKN-45 cells. (F-H) Wound healing assay showing that pretreatment with the Ca^2+^ inhibitor BAPTA/AM, the CaM inhibitor TFP, and the pan-Raf inhibitor LY3009120 abolished HRC-enhanced cell migration in AGS and MKN-45 cells. (I-L) Western blotting analysis of the protein levels of p-c-Raf, c-Raf, p-MEK, MEK, p-ERK, ERK, E-cadherin, N-cadherin, Vimentin, Snail, and Slug in GC cells transfected with Lenti-oeHRC and Lenti-oeControl, as well as cells treated with or without BAPTA/AM, TFP, and LY3009120. HRC, histidine-rich calcium binding protein; GC, gastric cancer; BAPTA/AM, membrane permeable 1,2-bis(o-aminophenoxy) ethane-N,N,N′,N′-tetraacetic acid; TFP; trifluoperazine; c-Raf, Raf-1 proto-oncogene, serine/threonine kinase; p-c-Raf, phosphorylated c-Raf; MEK, MAPK/ERK kinase 1; p-MEK, phosphorylated MEK; ERK, extracellular regulated kinase; p-ERK, phosphorylated ERK; CaM, calmodulin. Data are shown as the mean ± SD; ns: no significant difference; *P < 0.05, **P < 0.01, based on Student's t test.

To verify whether HRC-induced Raf/MEK/ERK phosphorylation is dependent on Ca^2+^/CaM signaling, GC cells were treated with BAPTA/AM (the intracellular Ca^2+^ chelating agent) and trifluoperazine (TFP), an antagonist of CaM. We demonstrated that BAPTA/AM and TFP pretreatment abolished HRC-induced cell invasion and migration (Fig. 5A-H). Furthermore, we also studied the effect of LY3009120, a pan-Raf inhibitor. As expected, the effect of LY3009120 was consistent with BAPTA/AM and TFP (Fig. 4A-H). We also found that pretreatment with BAPTA/AM, TFP, and LY3009120 prevented HRC-induced phosphorylation of Raf/MEK/ERK and EMT (Fig. 5I-L). These results suggested that HRC mediates the phosphorylation of Raf/MEK/ERK and EMT via Ca^2+^/CaM signaling.

### HRC modulates GC tumorigenesis and peritoneal metastasis (PM) *in vivo*

**Fig 6:**
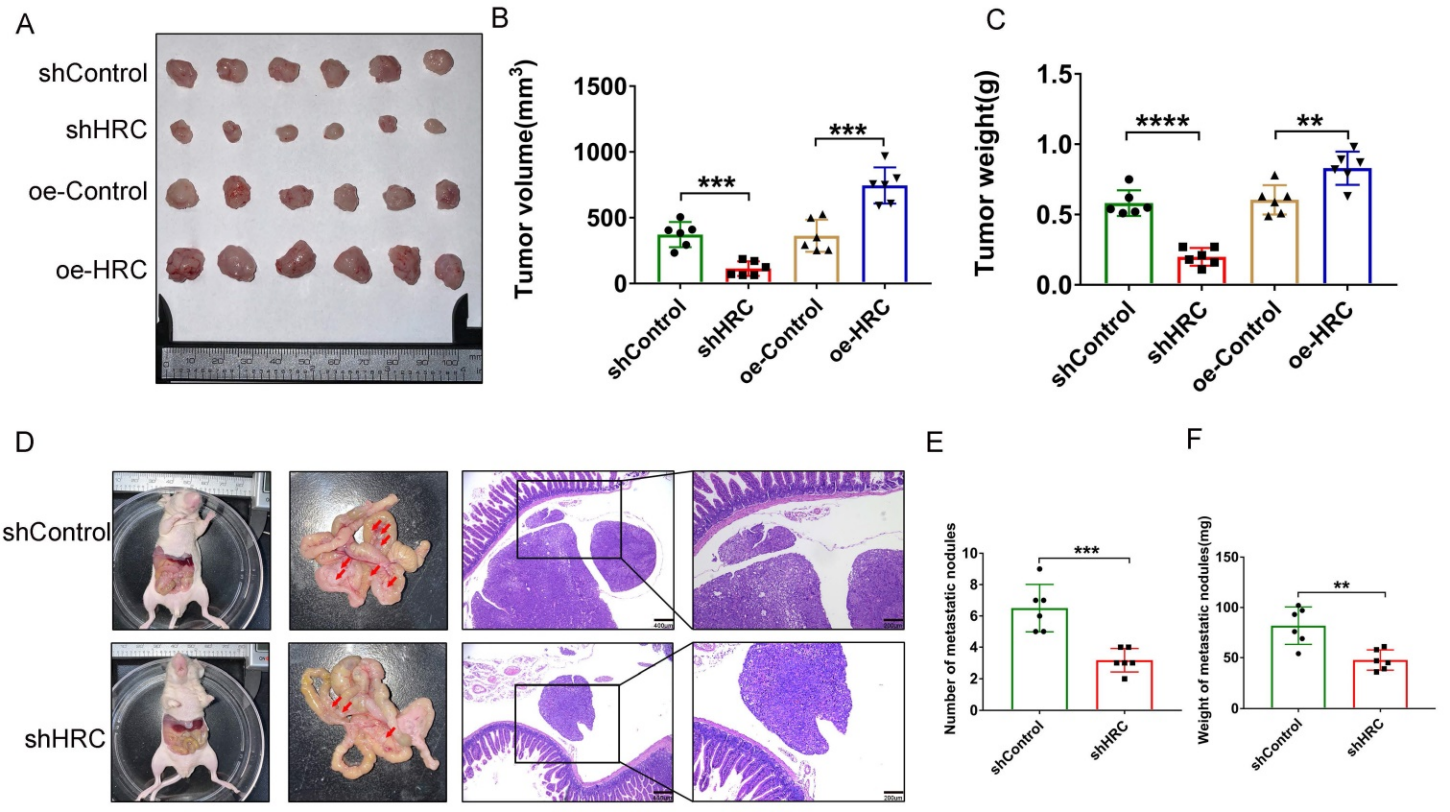
** HRC modulates GC tumorigenesis and peritoneal metastasis (PM) *in vivo.*** (A-C) Photographs of tumors excised from nude mice injected with AGS cells stably infected with Lenti‑shControl, Lenti-shHRC, Lenti-oeControl and Lenti-oeHRC lentiviral particles (n = 6 per group). Tumors were observed and recorded by tumor volume (B) and tumor weight (C). (D-F). Representative images of the macroscopic appearance of peritoneal metastatic nodules (red arrows) in nude mice treated intraperitoneally injection of AGS cells stably infected with negative control (NC) or HRC-shRNA lentiviral particles (N = 6 per group). The total number of peritoneal metastatic nodules in the respective groups (E). The total weight of peritoneal metastatic nodules in the respective groups (F). HRC, histidine-rich calcium binding protein; GC, gastric cancer; shRNA, short hairpin RNA. Data are shown as the mean ± SD; **P < 0.01, ***P < 0.001, ****P < 0.0001, based on Student's t test.

After fully understanding the effects of HRC* in vitro* on the regulation of the biological functions of GC cells, we tried to further clarify the role of HRC in promoting tumor growth *in vivo*. Thus, we constructed a subcutaneous xenograft model. We showed that the tumor size and weight decreased in the shHRC-AGS group but increased in the oe-HRC-AGS group (Fig. 6A-C). To determine whether HRC has an impact on GC PM, we constructed a peritoneal metastasis model. Compared with the AGS-shControl cells, AGS shHRC cells produced fewer and lighter PM nodules (Fig. 6D-F). In summary, these data showed that HRC is related to GC tumorigenesis and peritoneal metastasis *in vivo*.

## Discussion

Increasing evidence indicates that HRC exerts an important function in the regulation of intracellular Ca^2+^ uptake, storage, and release 4, 25. However, in the field of oncology, the biological role of HRC needs to be further investigated. Research on its role in tumor cells shows that it acts as an oncogene to regulate the biological behavior of liver and lung cancer cells 12, 13. GC is one of the most frequent causes of cancer-related deaths in the world 2. However, the effects of HRC on the biology of GC cells have not been reported. Therefore, we explored the regulatory effects of HRC on GC via *in vivo* and *in vitro* experiments.

In this study, we detected high expression of HRC in GC. Subsequently, we verified the relationship between the expression of HRC in the tumor tissues of patients with GC using a GC tissue microarray and revealed that high expression of HRC was a potential predictor of poor prognosis. To further clarify the function of HRC, we knocked down and overexpressed* HRC* in GC cells and found that knocking down *HRC* inhibited the invasion, metastasis, and proliferation of GC cells, while overexpression of *HRC* promoted cell metastasis, invasion and proliferation. These data indicated that HRC acts as an oncogene in GC.

HRC is a new type of intracellular calcium regulator. Calcium ions are intracellular signal transduction molecules, and evidence shows that they have an important regulatory effect on malignant phenotypes of tumor cells 26, 27. Although HRC's regulation of intracellular calcium is complicated and controversial, studies have shown that knocking down *HRC* can reduce the intracellular calcium concentration 10. GSEA in the TCGA-STAD cohort showed that *HRC* is important for the regulation of intracellular calcium. Therefore, we speculated that the effect of HRC on the biological phenotype of GC cells depends on its regulation of the calcium ion concentration in GC cells. Then, we found that knocking down *HRC* reduced the calcium ion concentration in GC cells. Moreover, we observed that the CaM protein level also decreased after* HRC* knockdown but increased after *HRC* overexpression.

Therefore, we identified Ca^2+^/CaM signaling regulated by HRC. It has been reported that HRC can regulate MEK/ERK and promote the progression of liver cancer 10. Similarly, we found that knocking down *HRC* in GC inhibited the activation of the Raf/MEK/ERK pathway. EMT plays a key role in the occurrence and development of tumors 28, including GC 29. The GSEA results for *HRC* also indicated that HRC is involved in EMT; therefore, we tested the relationship between EMT-related protein markers and HRC. Interestingly, we confirmed that knocking down *HRC* inhibited GC EMT. Treatment of GC cells with TFP and BAPTA/AM showed that HRC-induced Raf/MEK/ERK phosphorylation depends on Ca^2+^/CaM signals to regulate EMT in GC Finally, by constructing a subcutaneous tumor model and an abdominal cavity implant model, we found that HRC can promote tumor growth and proliferation in nude mice. Although we revealed the biological role of HRC in GC and its underlying mechanism, our experiments did not explore the exact mechanism by which HRC regulates the calcium ion concentration in GC cells.

## Conclusion

**Fig 7:**
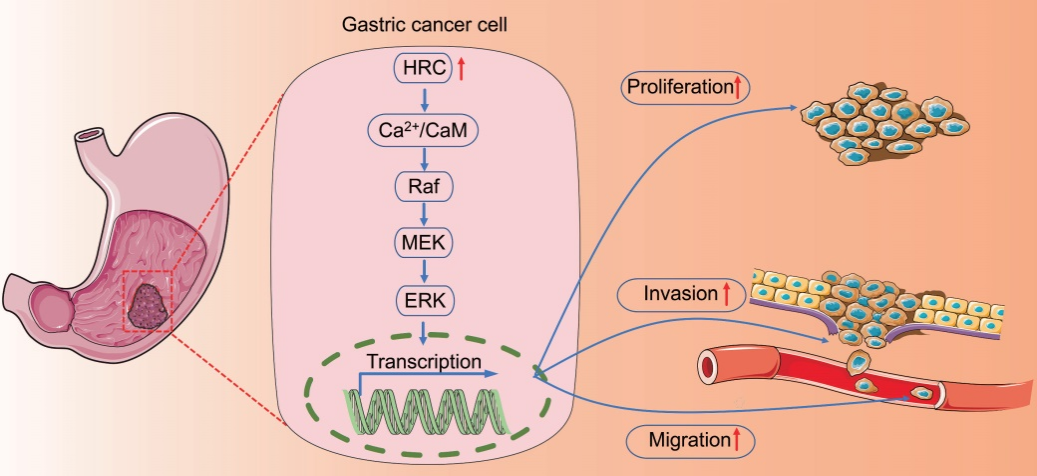
HRC regulates the level of Raf/MEK/ERK phosphorylation by changing the intracellular Ca^2+^ concentration and CaM protein level and ultimately affects the malignant phenotypes of gastric cancer cells.

In conclusion, we explored the expression and role of HRC in GC. We revealed that HRC might specifically promote cell migration, invasion, proliferation, and EMT via the Ca^2+^/CaM/Raf/MEK/ERK signaling pathway (Fig. 7). Therefore, HRC could be used as a potential prognostic marker and therapeutic target in GC.

## Supplementary Material

Supplementary figure.

## Figures and Tables

**Table 1:** Correlation analyses of HRC protein expression in relation to clinicopathologic variables of 85 GC patients.

Clinical factor		N	Low (HRC)	High (HRC)	*p* value
Age(year)					0.156
≤ 60		25	17	8	
> 60		60	29	31	
					
Gender					0.613
Male		63	33	30	
Female		21	13	8	
Unknown		1	0	1	
					
Tumor size(cm)					0.018*
≤ 5		39	27	12	
> 5		44	18	26	
Unknown		2	1	1	
					
Lymph node metastasis					0.065
No		22	16	6	
Yes		62	29	33	
Unknown		1	1	0	
					
T					0.009*
T1-T2		15	13	2	
T3-T4		56	25	31	
Unknown		14	8	6	
					
N					0.155
N0-N1		36	23	13	
N2-N3		48	22	26	
Unknown		1	1	0	
					
M					1
M0		83	45	38	
M1		2	1	1	
					
TNM stage					0.060
I+ II		32	22	10	
III + IV		45	20	25	
Unknown		8	4	4	

*p<0.05

## Abbreviations

HRC: Histidine-rich calcium binding protein
CaM: Calmodulin
EMT: Epithelial-mesenchymal transition
KEGG: Kyoto Encyclopedia of Genes and Genomes
OS: Overall survival
[Ca^2+^]_i_: intracellular Ca^2+^ concentration
PM: Peritoneal metastasis
GSEA: Gene set enrichment analysis

## References

[1] Necchi V, Candusso ME, Tava F, Luinetti O, Ventura U, Fiocca R (2007). Intracellular, intercellular, and stromal invasion of gastric mucosa, preneoplastic lesions, and cancer by Helicobacter pylori. Gastroenterology.

[2] Bray F, Ferlay J, Soerjomataram I, Siegel RL, Torre LA, Jemal A (2018). Global cancer statistics 2018: GLOBOCAN estimates of incidence and mortality worldwide for 36 cancers in 185 countries. CA Cancer J Clin.

[3] Chen W, Zheng R, Baade PD, Zhang S, Zeng H, Bray F (2016). Cancer statistics in China, 2015. CA Cancer J Clin.

[4] Arvanitis DA, Vafiadaki E, Sanoudou D, Kranias EG (2011). Histidine-rich calcium binding protein: the new regulator of sarcoplasmic reticulum calcium cycling. J Mol Cell Cardiol.

[5] Marchi S, Giorgi C, Galluzzi L, Pinton P (2020). Ca Fluxes and Cancer. Molecular cell.

[6] Monteith GR, Prevarskaya N, Roberts-Thomson SJ (2017). The calcium-cancer signalling nexus. Nat Rev Cancer.

[7] Xie R, Xu J, Xiao Y, Wu J, Wan H, Tang B (2017). Calcium Promotes Human Gastric Cancer via a Novel Coupling of Calcium-Sensing Receptor and TRPV4 Channel. Cancer research.

[8] Arvanitis DA, Vafiadaki E, Fan G-C, Mitton BA, Gregory KN, Del Monte F (2007). Histidine-rich Ca-binding protein interacts with sarcoplasmic reticulum Ca-ATPase. Am J Physiol Heart Circ Physiol.

[9] Gregory KN, Ginsburg KS, Bodi I, Hahn H, Marreez YMA, Song Q (2006). Histidine-rich Ca binding protein: a regulator of sarcoplasmic reticulum calcium sequestration and cardiac function. J Mol Cell Cardiol.

[10] Liu J, Han P, Li M, Yan W, Liu J, Liu J (2015). The histidine-rich calcium binding protein (HRC) promotes tumor metastasis in hepatocellular carcinoma and is upregulated by SATB1. Oncotarget.

[11] Liu J, Han P, Li M, Yan W, Liu J, He J (2015). Histidine-rich calcium binding protein promotes growth of hepatocellular carcinoma in vitro and in vivo. Cancer Sci.

[12] Xia S, Wu J, Zhou W, Zhang M, Zhao K, Tian D (2021). HRC promotes anoikis resistance and metastasis by suppressing endoplasmic reticulum stress in hepatocellular carcinoma. Int J Med Sci.

[13] Liu N, Li X, Fu Y, Li Y, Lu W, Pan Y (2021). Inhibition of lung cancer by vitamin D depends on downregulation of histidine-rich calcium-binding protein. J Adv Res.

[14] Lamouille S, Xu J, Derynck R (2014). Molecular mechanisms of epithelial-mesenchymal transition. Nat Rev Mol Cell Biol.

[15] Dongre A, Weinberg RA (2019). New insights into the mechanisms of epithelial-mesenchymal transition and implications for cancer. Nat Rev Mol Cell Biol.

[16] Mittal V (2018). Epithelial Mesenchymal Transition in Tumor Metastasis. Annu Rev Pathol.

[17] Dong J, Wang R, Ren G, Li X, Wang J, Sun Y (2017). HMGA2-FOXL2 Axis Regulates Metastases and Epithelial-to-Mesenchymal Transition of Chemoresistant Gastric Cancer. Clin Cancer Res.

[18] Bakir B, Chiarella AM, Pitarresi JR, Rustgi AK (2020). EMT, MET, Plasticity, and Tumor Metastasis. Trends Cell Biol.

[19] Lu W, Kang Y (2019). Epithelial-Mesenchymal Plasticity in Cancer Progression and Metastasis. Dev Cell.

[20] Wang C, Yang Z, Xu E, Shen X, Wang X, Li Z (2021). Apolipoprotein C-II induces EMT to promote gastric cancer peritoneal metastasis via PI3K/AKT/mTOR pathway. Clin Transl Med.

[21] Su C-C (2018). Tanshinone IIA inhibits gastric carcinoma AGS cells by decreasing the protein expression of VEGFR and blocking Ras/Raf/MEK/ERK pathway. Int J Mol Med.

[22] Jiang Y, Xie X, Li Z, Wang Z, Zhang Y, Ling Z-Q (2011). Functional cooperation of RKTG with p53 in tumorigenesis and epithelial-mesenchymal transition. Cancer research.

[23] Agell N, Bachs O, Rocamora N, Villalonga P (2002). Modulation of the Ras/Raf/MEK/ERK pathway by Ca(2+), and calmodulin. Cell Signal.

[24] Nussinov R, Muratcioglu S, Tsai C-J, Jang H, Gursoy A, Keskin O (2015). The Key Role of Calmodulin in KRAS-Driven Adenocarcinomas. Molecular cancer research: MCR.

[25] Park CS, Chen S, Lee H, Cha H, Oh JG, Hong S (2013). Targeted ablation of the histidine-rich Ca(2+)-binding protein (HRC) gene is associated with abnormal SR Ca(2+)-cycling and severe pathology under pressure-overload stress. Basic Res Cardiol.

[26] O'Grady S, Morgan MP (2021). Calcium transport and signalling in breast cancer: Functional and prognostic significance. Semin Cancer Biol.

[27] Roberts-Thomson SJ, Chalmers SB, Monteith GR (2019). The Calcium-Signaling Toolkit in Cancer: Remodeling and Targeting. Cold Spring Harb Perspect Biol.

[28] Brabletz S, Schuhwerk H, Brabletz T, Stemmler MP (2021). Dynamic EMT: a multi-tool for tumor progression. The EMBO journal.

[29] Seeneevassen L, Bessède E, Mégraud F, Lehours P, Dubus P, Varon C (2021). Gastric Cancer: Advances in Carcinogenesis Research and New Therapeutic Strategies. International journal of molecular sciences.

