# Supplementary material for: Histidine-rich calcium binding protein promotes gastric cancer cell proliferation, migration, invasion and epithelial-mesenchymal transition through Raf/MEK/ERK signaling
Source: J Cancer. 2022 Jan 4;13(4):1073–85. doi: 10.7150/jca.68403 (PMC8899383; doi:10.7150/jca.68403)
Supplement: Supplementary file 1 — Supplementary figure. [file jcav13p1073s1.pdf]

Supplementary materials

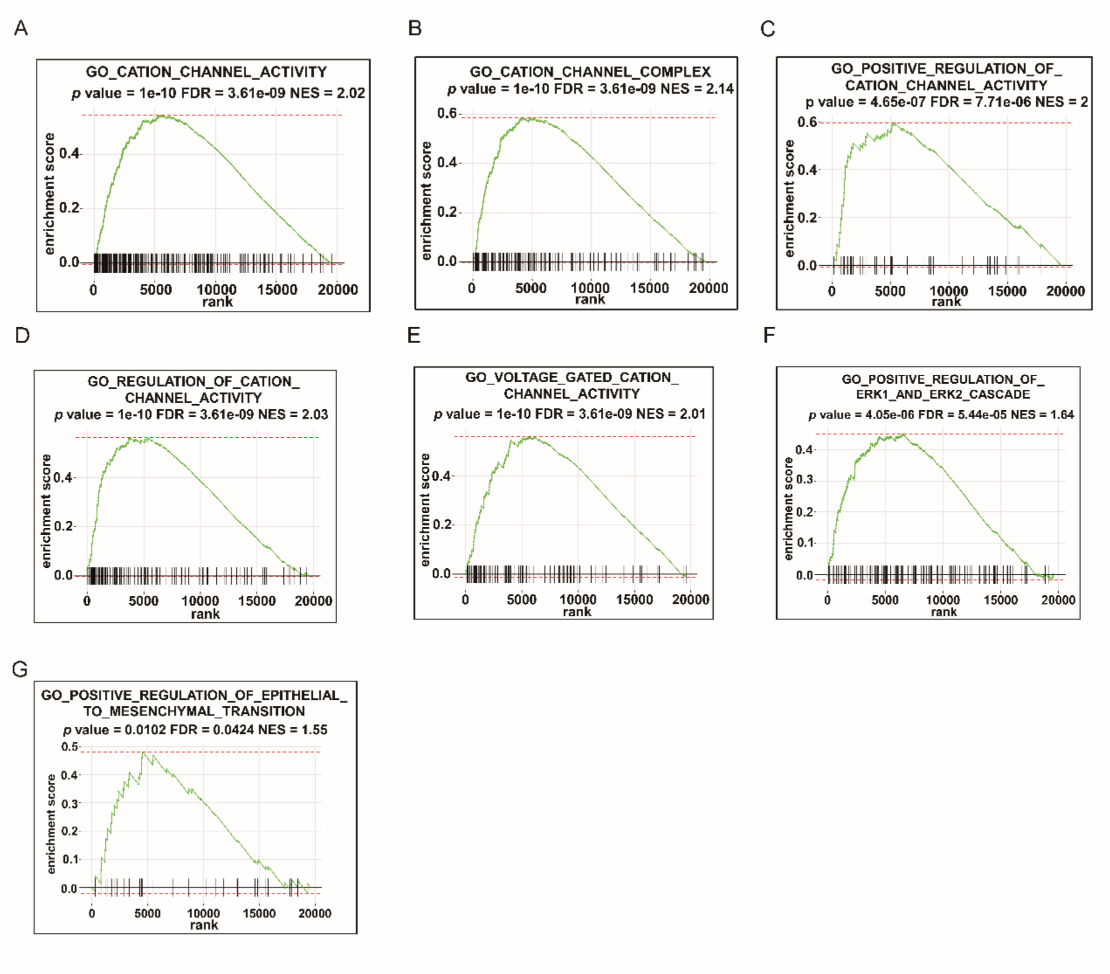

Fig. S1 GSEA enrichment analysis of HRC

(A-G) GSEA was performed using the TCGA-STAD cohort according to the correlation coefficient between the expression of HRC and all the other genes. HRC, histidine-rich calcium binding protein; GSEA, gene set enrichment analysis
